# Measuring access to and availability of outdoor recreational opportunities: One pixel at a time

**DOI:** 10.1016/j.landurbplan.2025.105445

**Published:** 2025-06-24

**Authors:** Allison Killea, Jeremy Baynes, Donald Ebert, Anne Neale

**Affiliations:** a ORAU National Student Services Contractor supporting US EPA, Research Triangle Park, NC, USA; b US EPA, Office of Research and Development, Research Triangle Park, NC, USA

## 1. Introduction

It is well accepted that outdoor physical activities are good for our mental and physical health along with the social cohesion and emotional wellbeing we often experience in outdoor places. Many studies have examined the health and wellbeing benefits of recreating outdoors, especially in natural areas. Within the context of this paper, ‘natural areas’ includes areas in a natural state as well as semi-natural areas (e.g., planted and managed vegetation and water features). When compared with indoor activity, outdoor activity in a natural or green environment frequently leads to better health outcomes. Outdoor recreation, in green or more natural environments, is consistently correlated with decreased illness, lower rates of violence, greater physical activity rates, and better mental health within the population (Coombes et al., 2010; Jennings & Bamkole, 2019; Kondo et al., 2018; Twohig-Bennett & Jones, 2018; World Health Organization, 2016). Proximity to a city park and surrounding greenness were associated with better birth outcomes (Grazuleviciene et al., 2015; Villeneuve et al., 2022). Time spent recreating in green space has been associated with improved mental health and lower obesity rates in children (McCurdy et al., 2010) and access to walkable greenspace has been linked to the longevity of senior citizens in urban environments (Takano et al., 2002; Yuan et al., 2021).

Urban and other developed areas face unique challenges in providing adequate outdoor recreational opportunities. Obesity and many associated health complications have been found at higher rates in urban environments that fail to encourage pedestrian and other physical activities (Congdon, 2019). One study of ten parks in five United States (U.S.) cities found that time spent in a park accounted for 50 % of vigorous physical activity time for those within a 0.5 mile walk of the park and 16 % for those living within one mile of the park (Han et al., 2013). The United Nations (2019) estimates 55 % of the global population lives in urban areas and over 80 % of Americans reside in U.S. Census-defined urban areas (U.S. Census Bureau, 2021). In densely populated urban centers, outdoor recreational areas may be smaller and less natural compared to rural or suburban areas. However, urban areas can take advantage of their denser transportation networks, sidewalks, and public spaces to promote pedestrian access to outdoor physical activities (Han et al., 2013). For the purposes of this study, public outdoor recreation areas (PORAs) are managed spaces that are available for public use and are typically dominated by at least some natural and semi-natural features. We chose to use the term ‘PORAs’ instead of ‘parks’ because ‘parks’ are loosely defined, many PORA’s do not include the term ‘park’ in their designation, and because the definition of ‘parks’ is often in the eye of the reader. For some ‘park’ conjures visions of Yellowstone National Park, and for others, a skate park in their city’s downtown district. Outdoor recreation activities occurring in PORAs range from extremely physical (e.g., trail running, cross-country skiing, tennis, basketball) to more passive (sightseeing, painting, viewing nature, picnics). While PORAs with little or no natural features (e.g., basketball courts, skate parks) may not convey all the benefits as PORAs with more natural features, these areas are important to consider in conjunction as they both promote social cohesion and provide opportunities for physical activity.

Many methods have been devised to quantify who does or does not have sufficient access to PORAs. Some assesments focus on parks alone, others consider all urban greenspace, but the general inquiry remains the same: are people able to recreate outdoors? Ekkel and de Vries (2017) detailed that most of today’s metrics can be considered either “residential proximity accessibility metrics” or “cumulative opportunity metrics”. The former is designed to identify people within a specified distance of an area of interest. A suitable distance threshold is a major decision in measuring access that is often debated (Wang et al., 2021); however, walkable distances are commonly between 300 and 800 m (Ekkel & de Vries, 2017; Walk Score, 2022). Distance may be calculated using a simple radius (California Department of Parks and Recreation, 2022; Liu et al., 2022; Stanford University et al., 2025) or more complex walkable routes derived from road network or landscape data (Trust for Public Land, 2023; U.S. Environmental Protection Agency, 2015). People who are close enough to a qualifying PORA are considered as having access, often without regard to the size or total number of PORAs relative to the density of the population. Cumulative opportunity metrics calculate the total area of PORAs available relative to the population within a given reporting unit, such as a census tract or city boundary (California Department of Parks and Recreation, 2022). This method may not account for the accessibility of that space to the target population. While some efforts, like California’s Parks for All Californians program, perform both types of analyses, they are separate measures rather than a cohesive metric that considers accessibility and availability in the same measure (California Department of Parks and Recreation, 2022; Coutts et al., 2010). Liu et al. (2022) proposed a gravity model that combines accessibility and availability to urban greenspace to address this gap; however, it uses a radius around each pixel in the calculation rather than incorporating a transportation network and barriers, such as an uncrossable river. Zhang et al., (2011) offer a population-weighted distance model that addresses the spatial relationship between population density and PORA sizes and distribution, but again, it relies on broad estimates of proximity (Euclidean distance buffers) to population density and uses a centroid within a reporting unit (i.e., census blocks) rather than being pixel-based. To our knowledge, there is no existing measure that is network-based and that integrates pixel level availability and accessibility to PORAs and is broadly applicable across PORA types, settings, sizes, etc.

It is critical that planners and researchers have adequate information to better understand both PORA accessibility and availability. In the context of this study, we refer to the accessibility of a PORA based on a measure of proximity or nearness to a population along walkable routes and availability denotes the area of PORA provided per person who can access it. Although accessibility and availability of PORAs can be considered separately, it can be useful to assess them in combination. PORAs are congestible; they can only be utilized by so many people at one time. For planning and management purposes, it is important to know which populated areas are within walking distance of a PORA and how many people are relying on that shared resource. Understanding the spatial variations in accessibility and availability to PORAs is key to planning for future investment and improvement to attain adequate access to outdoor recreation areas.

We have devised a new method, tool, and data set that measures both accessibility and availability of PORAs. We applied this method to the conterminous United States (CONUS). For the purposes of our study, we measured PORA access as living within an 800 m, or roughly 10-minute, walk of a PORA. We recognize that an assessment of PORAs within a 10-minute walk in sparsely populated areas, while interesting as a comparison and illustrative of the paucity of PORAs within walking distances in these areas, is perhaps less relevant. In these low population density areas where driving to amenities is more common, placing PORAs within 800 m of everyone is not practical. Findings from the 2017 National Household Travel Survey confirmed that rural households are much more dependent on private vehicle travel (McGuckin & Fucci, 2018). However, the method and tools we have developed should be applicable using other travel distances (e.g., short drive), transportation networks (e.g., local sidewalks), and amenities (e.g., local parks or greenspaces).

Once published, the data and the code will be available through EnviroAtlas (https://epa.gov/enviroatlas), an online resource providing geospatial data and tools relating to the benefits humans receive from ecosystems (i.e., ecosystem goods and services).

## 2. Methods

We first describe the metric and the method/tool we developed to assess PORA accessibility and availability within walking distance. We then describe the data inputs and tool parameters we used to apply the metric CONUS-wide. We also illustrate how this metric can be used in a community PORA assessment using two different sized cities as examples. Finally, we validate our results by comparing them to other methods.

### 2.1. Metric description

**Fig. 1.**
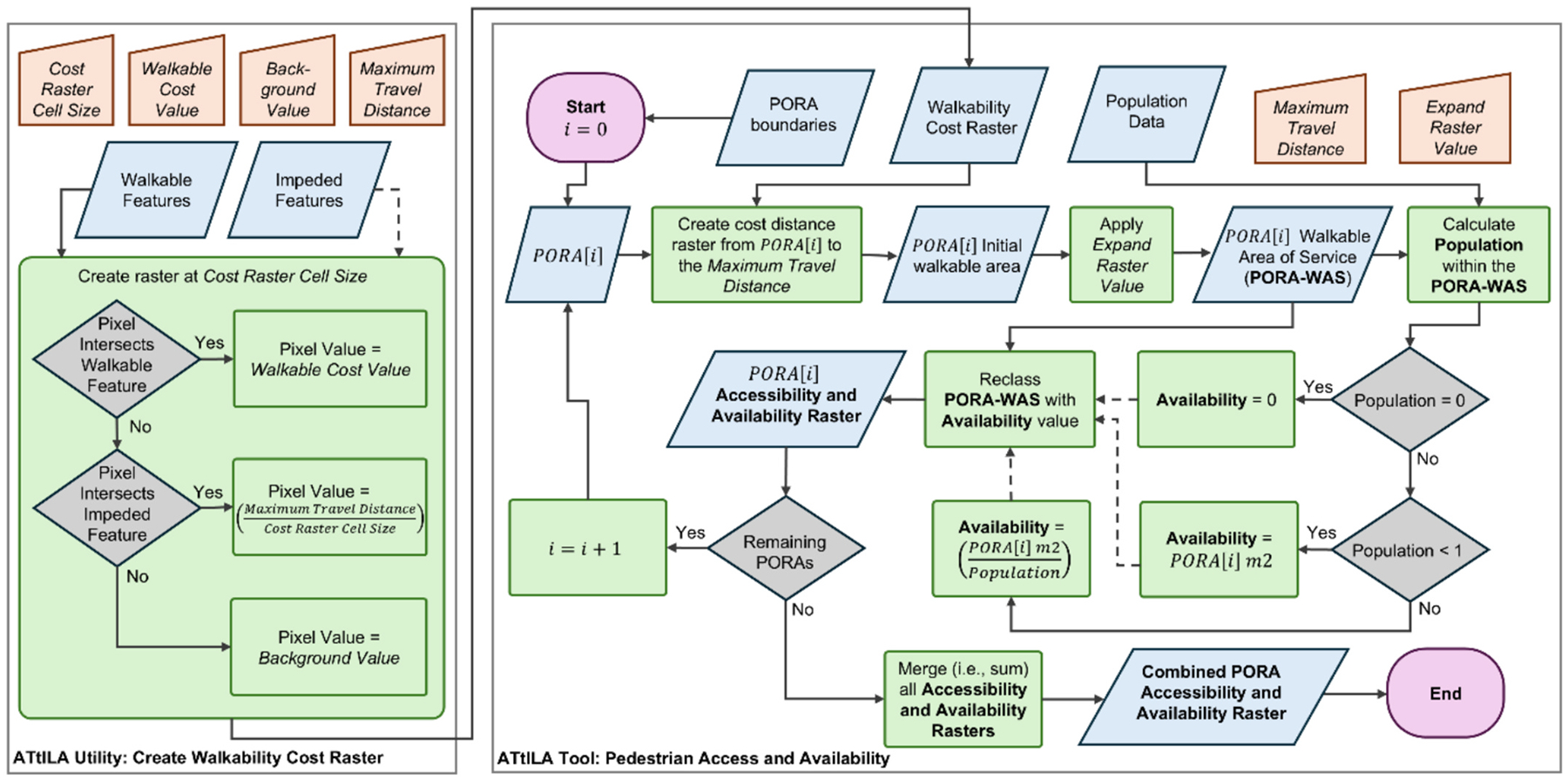
Workflow for measuring PORA accessibility and availability.

We define accessibility to a PORA as a person being within a specified distance of a PORA and availability as the area of PORA provided per person within that distance. For our study, we were focused on accessibility and availability of PORA’s within walking distance which raised the challenges of determining which areas were walkable and how many people lived within that walkable area. We used a multi-step, raster-based approach to measure accessibility and availability of PORAs (Fig. 1), details are described in Sections 2.1.1 – 2.1.4.

#### 2.1.1. Accessibility

Identifying regions within a specified distance along a road network can be accomplished using a traditional GIS vector service area analysis where information such as direction of travel and speed limits can be incorporated to model distances more precisely. However, this type of analysis requires users to either create complex network datasets or use a commercial service for a fee, neither of which are ideal for modelling over a large area (e.g., CONUS). A cost surface (i.e., raster) approach offers an alternative to vector service analyses. A cost surface is a gridded digital representation of ground conditions where each cell (i.e., pixel) is assigned a value representing the cost (e.g., time, distance, money) to travel across that cell. Travel over specific land cover types can be prioritized or avoided by adjusting the cost for the cells that overlay with those features.

We assume people will tend to follow existing transportation corridors (e.g., walkable roads) but may deviate across non-developed terrain for short distances. For example, pedestrians might briefly cross grassy strips along the shoulder of a walkable road to access a PORA but would not likely walk long distances on such surfaces. We also assume some areas are not suitable for pedestrian use and should be avoided.

**Fig. 2.**
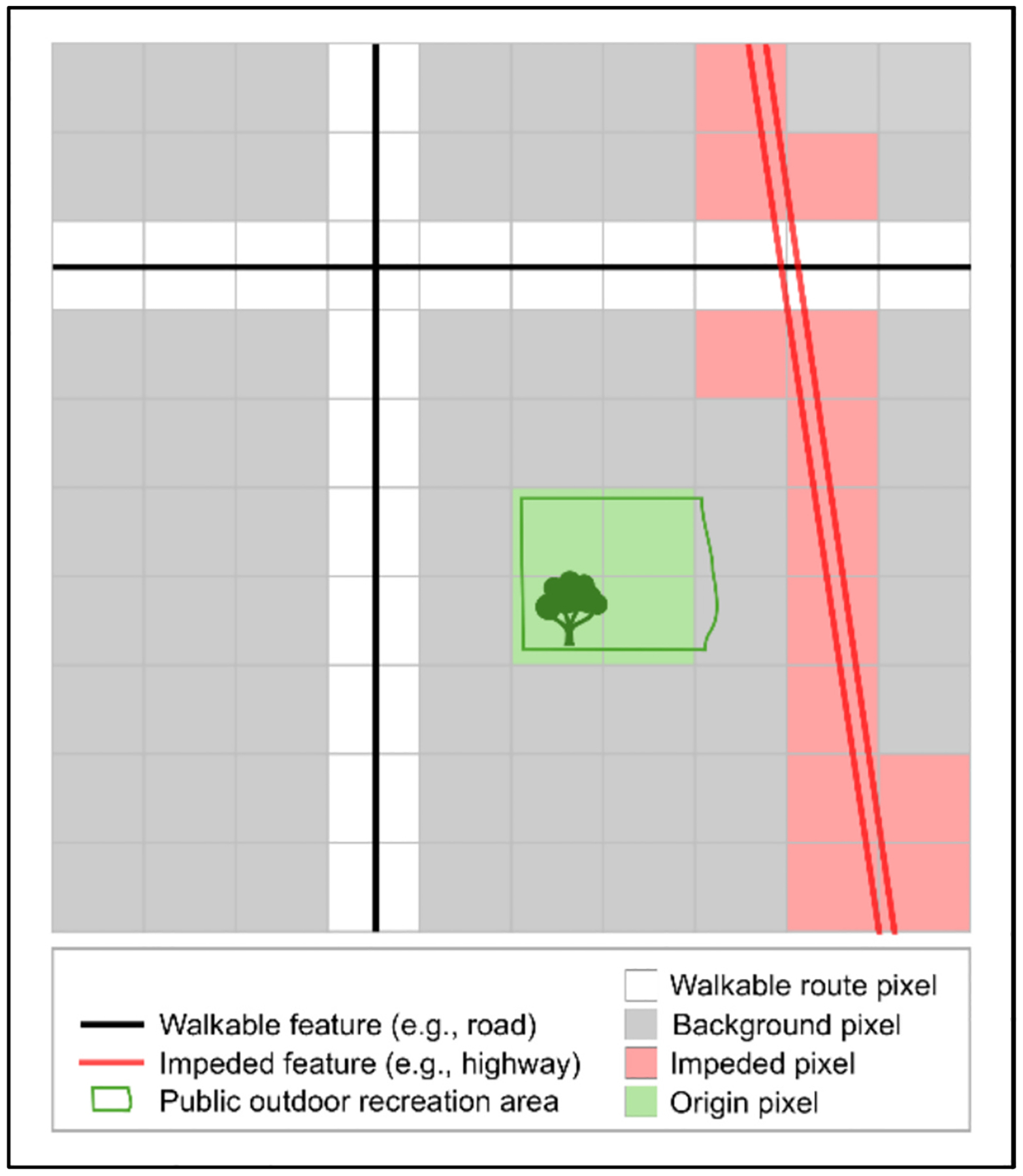
Walkability cost raster. White indicates walkable routes (i.e., low cost), gray indicates background value (i.e., higher cost), red indicates impeded surfaces (i.e., highest cost). Walkable routes allow pedestrians to continue across impeded surfaces (e.g., underpass or bridge). PORAs that do not intersect or are not adjacent to a walkable route can have accessibility through one or more background pixels.

To represent these differing landscapes, we create a cost raster with three pixel values: walkable, background (i.e., any surface not known to be either walkable or impeded), and impeded/unsafe for pedestrian use (Fig. 2). Pixels that intersect a walkable feature are assigned the ‘walkable’ value (e.g., a value of one). Pixels that intersect an impeded feature are set to a value that ensures that no travel is allowed beyond that pixel. Pixels that intersect both a walkable feature and an impeded feature are assigned the walkable value, allowing pedestrians to continue rather than be obstructed. Examples of this include roads over culverted streams, bridges over water, and small roads passing under major freeways. All other pixels are assigned a background value. A background value allows travel off walkable paths to reach a destination but reduces the total travel distance allowed because of the increased cost. Increased cell values in the cost raster shortens the distance a pedestrian can move to account for the increased difficulty of passing through those areas. This represents typical pedestrian behavior and ensures that PORAs do not need to intersect a walkable road to be considered accessible.

**Fig. 3.**
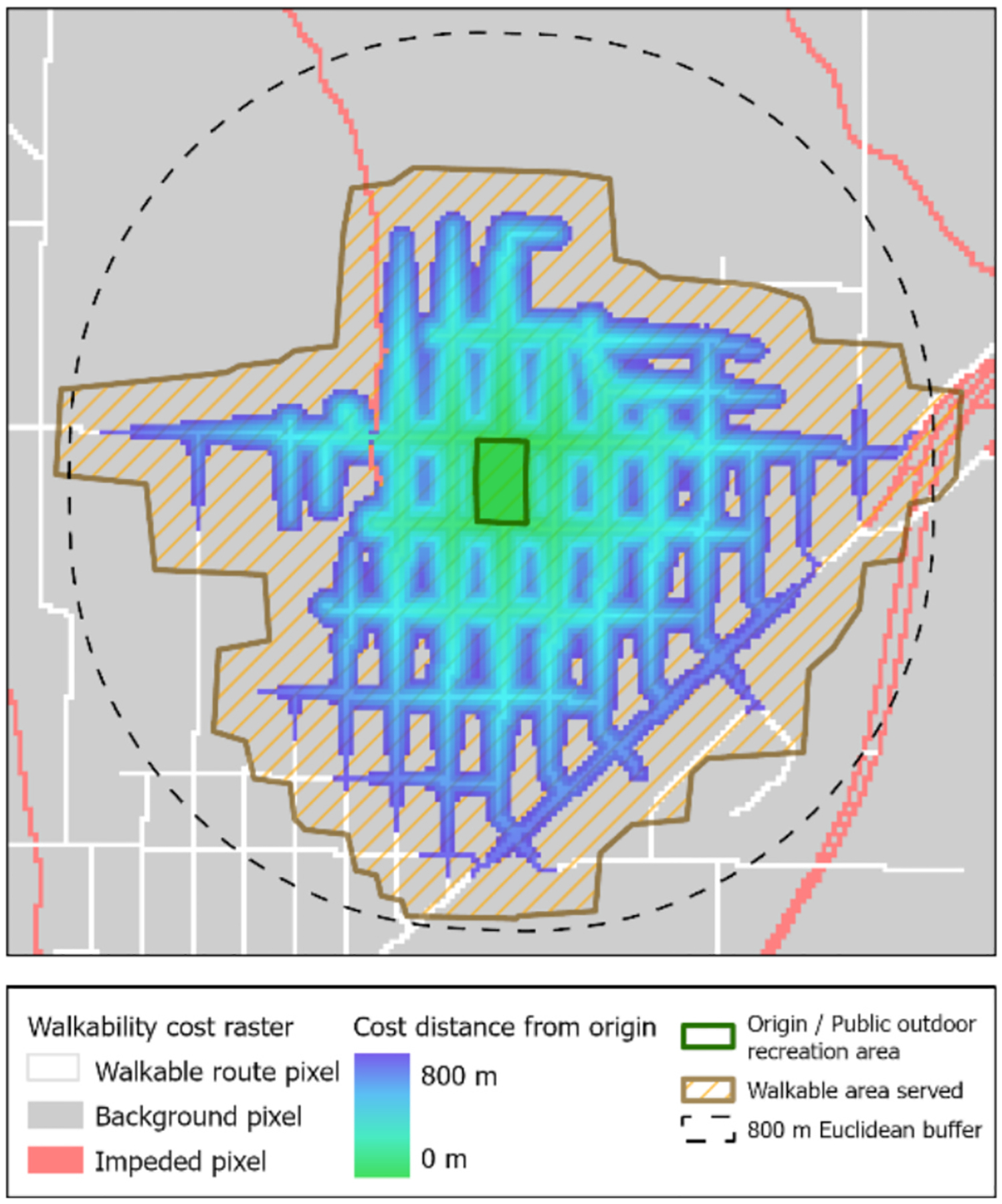
Map of PORA-WAS. The PORA-WAS is the accumulated cost distance from origin (i.e., initial walkable area) plus an 8-pixel expanded area. For reference the dashed line illustrates the area that would be included using a Euclidean distance for the same walking distance.

Based on the cost raster, we identify pixels that intersect a PORA boundary as the origin and determine all pixels within a maximum accumulated travel distance from the origin (Fig. 3). This represents the initial walkable area for each PORA. The initial walkable area naturally extends along walkable road networks while potentially omitting some residential areas alongside and between roads (Fig. 3). For example, in a gridded city layout, the initial walkable area may extend along all four roads of a city block but would not include the interior of that block. Furthermore, there is a slight discrepancy between measuring distances as a straight vector line and as the distance between centroids of adjacent raster pixels. Expanding the initial walkable area by a fixed number of pixels fills many of these small areas and offsets the underestimation of walkable areas measured using raster distances. This expanded area represents the raster-based walkable area served (WAS) for each PORA (PORA-WAS) (Fig. 3).

#### 2.1.2. Availability

To measure availability, we determine the size (in square meters) of each PORA and count the population within each PORA-WAS. PORA-WAS with populations of zero are considered to provide zero square meters of PORA within walking distance; therefore, we set availability to zero. For PORA-WAS with less than one person (but greater than zero), we set availability as the area of the PORA. Estimated population values of less than one person can occur in areas where population density is low (e.g., rural areas). For PORA-WAS with population greater than or equal to one, we calculate availability by dividing the PORA area by the population in the PORA-WAS. We reclassify the value of all cells in the PORA-WAS raster with the PORA availability value (i.e., square meters per person of PORA). This represents the accessibility and availability for an individual PORA.

#### 2.1.3. Combined accessibility and availability

**Fig. 4.**
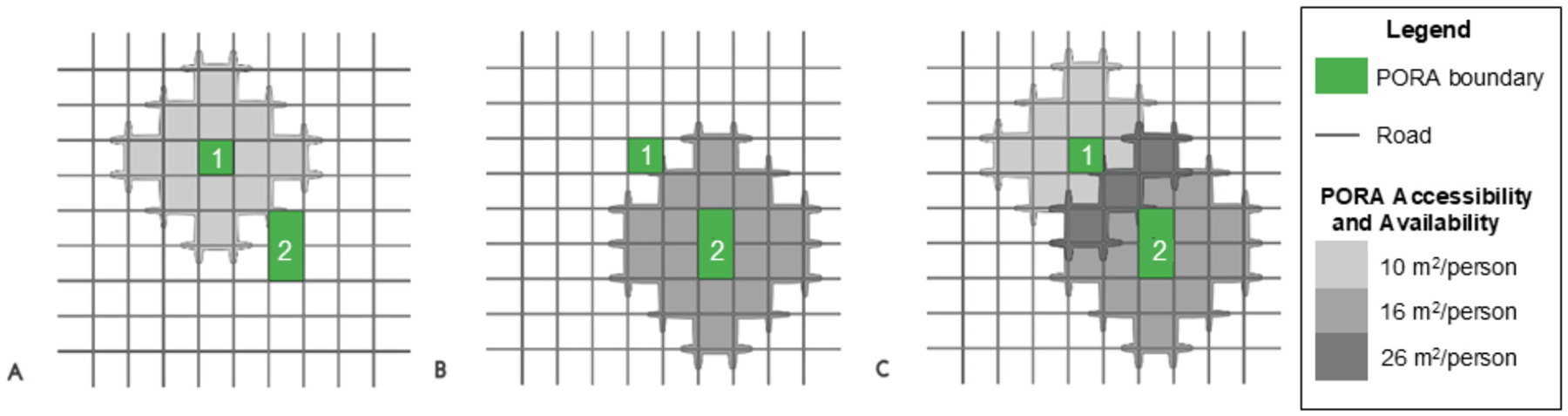
A simulated example of urban PORAs and road network. In panel A, we assume PORA 1 is 1,000 m^2^ and 100 people live in the WAS (light gray area). In panel B, we assume PORA 2 is 2000 m^2^ and 125 people live in the WAS (medium gray area). Panel C demonstrates the cumulative effect for the people who live in the overlapping, darkest gray area; they have the combined availability of 26 m^2^/person. Example is not to scale.

After determining accessibility and availability for each PORA (Fig. 4A–B), we create a combined PORA accessibility and availability raster by summing all individual PORA results into a single layer (Fig. 4C). This creates a continuous raster where the value of a pixel represents square meters of PORA available per person. This step accounts for people having access to multiple PORAs.

#### 2.1.4. GIS toolbox

The methods described in 2.1.1–2.1.3 have been implemented in the EPA’s Analytical Tools Interface for Landscape Assessments (ATtILA) (U.S. Environmental Protection Agency, 2023). ATtILA is a custom ArcGIS Pro toolbox that calculates many landscape and landscape/human interaction metrics. The workflow is available in ATtILA as a two-step utility and tool (Fig. 1). The Create Walkability Cost Raster (CWCR) utility creates a cost raster that can be used as an input to the Pedestrian Access and Availability Tool (PAAT). We opted to make this a two-step process to give users the option of bypassing our cost raster creation step if they chose to utilize a different weighting scheme (e.g., more than three weights).

The CWCR utility allows users to select multiple walkable and impeded features (e.g., roads, sidewalks, water), adjust the costs (i.e., weights) for those features, set a background cost, and set the cell size for the cost raster. The PAAT iterates over each feature (e.g., PORA) to measure PORA accessibility and availability for a given area. Required inputs for the PAAT are a polygon layer that defines PORAs, a cost raster, and a polygon or raster population dataset. Users can adjust the parameters for maximum travel distance and the number of pixels to expand the initial walkable area. PORAs that are too small or irregularly shaped to intersect the centroid of a cost raster cell are omitted. The resulting product is a floating-point raster where each grid cell represents the square meters of PORA per person available. Values of zero represent areas with no population that are within a PORA-WAS and null values represents areas without PORA access. Detailed usage notes and syntax are available in the GitHub repository wiki for ATtILA (https://github.com/USEPA/ATtILA2/wiki).

### 2.2. Applying our metric

We relied on nationally consistent datasets to measure accessibility and availability of PORAs for the conterminous United States (Table 1).

#### 2.2.1. PORA boundaries

For PORA boundaries, we used the Protected Areas Database US – Accessible Recreational (PAD-US-AR), a curated dataset based on the USGS Protected Areas Database (PAD-US) to identify publicly accessible outdoor recreation areas (Browning et al., 2022; U.S. Geological Survey, 2024). PAD-US serves as an authoritative national dataset of terrestrial and marine protected areas across the entire United States dedicated to identifying lands preserved for biodiversity, recreation, and culture (U.S. Geological Survey, 2024). PAD-US includes data from Federal agencies, State agencies, and non-governmental organizations and in more recent releases has incorporated data from Trust for Public Land’s ParkServe, The Nature Conservancy, and Ducks Unlimited. PAD-US may serve a range of natural and cultural uses including, but not limited to, recreation (U.S. Geological Survey, 2024). Browning et al. (2022) identified areas likely accessible for recreation using PAD-US V2.1 based on a consensus between the authors and multiple outdoor recreation specialists. The rationale and full documentation of their decisions are explained in Browning et al., (2022). In short, areas identified as proclamation lands, marine areas, or closed access in PAD-US were excluded; areas managed by 19 specific organizations or types (e.g., Department of Energy, Department of Defense, non-governmental organizations, American Indian Lands) with unknown access were excluded; and state trust lands in 16 US States were excluded (Browning et al., 2022). The PAD-US-AR dataset aligned well with the requirements and goals of our analysis, and we chose to use their data without further considerations while acknowledging the limitations of curating compiled data from multiple sources with imperfect attributes.

We spatially dissolved PAD-US-AR polygon features and removed any identical polygons to avoid overlapping or duplicate boundaries. PORAs immediately adjacent, but on either side of an administrative boundary (e.g., state, county), were dissolved into one polygon. This step corrected for instances such as national parks being artificially split into segments corresponding to the states they span as well as eliminating slivers that arose from a discrete state attribute being assigned to each PAD-US-AR polygon.

#### 2.2.2. Walkability

We used several datasets to identify routes likely to be suitable or unsafe for pedestrian use. For instance, water is a deterrent to pedestrian movement. Data from USGS’s National Hydrography Dataset (NHD) Plus High Resolution (HR) was used to identify water features. NHDPlus HR is a publicly available dataset describing water bodies and flows across CONUS as well as Hawaii, Alaska, and several U.S. Territories (U.S. Geological Survey, 2022). These features in the NHDPlus HR dataset were considered unsafe for pedestrian use in our cost raster:

1. Oceans
2. Bays/Inlets
3. Flumes
4. Rapids
5. Areas of complex channels
6. Rivers/Estuaries/Streams
7. Lake/Pond/Reservoirs
8. Canals/Ditches
9. Connectors

Roads can be either walkable or unsafe for pedestrian use. Roads were identified using the ArcGIS StreetMap Premium North America 2021 Release 1. StreetMap Premium contains enriched street data covering the extent of CONUS as well as much of the globe. The dataset is licensed and maintained by ESRI and based on reference data from commercial providers such as HERE and GeoTechnologies, Inc (Esri, 2021). StreetMap Premium assigns attributes with each road feature that enables differentiation between pedestrian-friendly and unsafe roads. Certain roads are not suitable for pedestrian use. Roads in StreetMap Premium (Esri, 2021) were considered unsafe for pedestrian use if they met any one of the following criteria:

1. A function class less than 3 (i.e., roads that allow for a high speed and volume of movement between and through cities or interconnect such roads)
2. Speed limits greater than 54
3. Restricted against pedestrians
4. Tollways
5. Ferry routes
6. Entrance/exit ramps
7. Roads with controlled access

Roads not identified as unsafe for pedestrian use were considered “walkable roads”. This may result in more roads being considered walkable than are walkable in practice and in turn we may overestimate actual walkability along some routes. We erred in this direction to be confident in our estimate of areas beyond walking distance of a PORA.

We did not include any sidewalk or walking path data as to our knowledge, there is no such dataset available that is nationally consistent and reliable. Furthermore, we are aware many PORAs are bounded by fences that make them inaccessible along the entirety of their perimeters. While it is possible to include known barriers as impeded when developing the cost raster, identifying those barriers is not practical for a CONUS-wide effort. Others have modeled PORA entrances with road or path intersections (Trust for Public Land, 2023; Wang et al., 2021), but we opted not to do this as there are also many examples of PORAs that are entirely accessible along their perimeters (e.g., the National Mall in Washington DC). Though we did not explicitly model park entrances or barriers, using higher weights for background and impeded cells in the cost raster mimics these other efforts by prioritizing travel where walkable paths are near PORAs.

#### 2.2.3. Population

The 2020 United States Environmental Protection Agency’s (EPA) EnviroAtlas Dasymetric Population Map for CONUS was used to determine population within each PORA-WAS. It is a 30-meter resolution gridded estimate of population density based on the 2020 US Census Survey block data through intelligent dasymetric mapping. Population is often distributed heterogeneously throughout census blocks and this product, based mostly on land use and land cover data, provides a more accurate and precise estimate of where people live on the landscape (Baynes et al., 2022).

#### 2.2.4. ATtILA parameters

The ATtILA Toolbox v3.0 was used to calculate accessibility and availability for CONUS with the input data described above (Table 1) and with the parameter settings listed in Table 2.

We chose to use a maximum distance of 800 m. This distance is approximately a ten minute walk and is used by many similar analyses as a general rule of thumb for how far someone will walk to a PORA (National Recreation and Park Association, 2016; Telega et al., 2021; U.S. Green Building Council, 2021; Yang & Diez-Roux, 2012; Zuniga-Teran et al., 2016).

The cell size of the cost-distance raster is a major factor in how well a cost-raster and vector service area align (Choi et al., 2014; Delamater et al., 2012; Mulrooney et al., 2017). A smaller cell size reduces instances where two roads exist within the same cell and better represents the vector network but increases computational requirements (Delamater et al., 2012). Based on this tradeoff, we chose a 10 m cell size.

The ATtILA CWCR tool was used to create a cost raster where water features and road segments identified as unsafe for pedestrian use were set to a value of 80 (i.e., maximum travel distance divided by raster cell size), walkable roads were assigned a value of 1, and all other pixels were considered background and assigned a value of 10. These values represent typical pedestrian behavior where people are more likely to stay on walkable paths, travel short distances over other surfaces, and avoid unsafe or inaccessible areas.

We tested five options (0, 3, 5, 8, and 10 pixels) for the Expand Raster Value parameter and selected 8 pixels for this analysis (see section 2.4.2). The ATtILA PAAA tool was used to iterate over each PORA in PAD-US-AR and produce the accessibility and availability metric for CONUS. For improved processing efficiency, the PAAA tool was applied for consecutive batches of 1,000 PORA features and the results of all batches were then merged.

### 2.3. PORA assessment for CONUS

We are not aware of any widely agreed upon standard for how much space within a PORA an individual generally needs to gain minimal recreational benefits. Individual and community needs vary, as do the types of spaces and their many effects, making it hard to prescribe an amount (Ekkel & de Vries, 2017). While our goal is not to recommend an amount of space, we did select an upper and lower benchmark against which to apply our results. Of the many thresholds proposed in the literature, LEED v4.1 requires that 11.25 square meters of greenspace be provided per person within a city or community to meet their sustainable city certification (U.S. Green Building Council, 2021). The National Resource Center for Health and Safety in Child Care and Early Education recommends a minimum 3 square meters of outdoor play space to prevent collisions among active children at play (American Academy of Pediatrics, 2023). We reclassified our availability and accessibility results into two binary raster datasets to identify areas below the 3 and 11.25 square meter thresholds (pixel values of 1 < threshold; 0 ≥ threshold). Likewise, we created a binary raster dataset to identify all areas with either no access or zero square meters of availability.

#### 2.3.1. Summary units

To better understand PORA accessibility and availability across CONUS, we summarized our results by H3 hexagons (Uber Technologies, 2023). Summarization by a reporting unit is helpful to illustrate patterns in raster data across large areas such as CONUS. Unlike typical administrative units which vary considerably in size and shape, hexagons provide a consistent geographic unit allowing users to assess and combine measurements (e.g., percent imperviousness and number of people with PORA access) and compare conditions across space in a consistent manner. H3 is a 16-level nested hexagonal global grid system and we selected H3 Resolution 9 for this analysis. We chose level-9 because they are small enough to detect differences across neighborhoods and communities and also feasible based on computing requirements for CONUS processing. At this resolution, there are approximately 75 million hexagons at an average size of 105,332 m^2^ (Uber Technologies, 2023) in CONUS. All H3 hexagon statistics were calculated using Esri ArcGIS Pro Zonal Statistics tools.

Total population was determined for each hexagon using a zonal sum of the EPA dasymetric population raster. Population with no PORA access, population with less than 3 m^2^ of availability, and population with less than 11.25 m^2^ of availability were determined using zonal sums of the EPA dasymetric raster after masking by the respective binary accessibility and availability results.

We also calculated percent imperviousness using a zonal mean of the 2019 National Land Cover Database (NLCD) Impervious Descriptor layer (Dewitz, 2021) for each hexagon. The NLCD Impervious Descriptor is a measure of what proportion of each 30-meter pixel is detected as impervious surface based on corresponding Landsat imagery. The percent impervious measure was used to identify hexagons with less than 70 %, between 70 and 85 %, and over 85 % impervious surface. The logic behind selecting these thresholds is that areas with greater impervious surface have less incidental greenspace (e.g., back yards) available for outdoor recreation and therefore may have even greater need for PORAs. These values were selected to illustrate how our data can be used to conduct a comparative analysis between areas with differing amounts of impervious surface and are not intended as recommended thresholds of impervious surfaces.

#### 2.3.2. Example case studies

We illustrate our results for two cities, New York City, NY and Sioux City, IA.

### 2.4. Metric validation

#### 2.4.1. Comparison to other measures

We compared our estimate of areas within walking distance of a PORA to values reported from two different resources: California Parks for All (California Department of Parks and Recreation, 2022) and ParkServe (Trust for Public Land, 2023). We chose these two resources because they used roughly comparable PORA data and maximum walking distance. California Parks for All uses a 0.5 mile (~804 m) radius rather than walkable routes from each PORA (California Department of Parks and Recreation, 2022). See Fig. 3 for an approximation of the 0.5-mile radius. ParkServe approximates a 10-minute walk using 0.5 mile distance along walkable routes to park access points to establish their walk service area for each PORA (Trust for Public Land, 2023). We compared PORA area and PORA-WAS for two different sized urban areas, New York City, NY and Sioux City, IA along with two counties in California, Orange and San Francisco. For these comparisons, we excluded the area of the PORA/park from all service area measurements.

#### 2.4.2. Comparison of PORA-WAS and vector service areas

We compared our raster-based PORA-WAS with a traditional vector-based service area using Esri’s ArcGIS Pro and ArcGIS Online. Because of the costs of using a commercial service, we limited our comparison to 1,000 randomly selected PORAs. The ArcGIS Pro tool, Make Service Area Analysis Layer was used to create an analysis layer for walking distance with a cutoff at 800 m. We accepted all default parameters available in the Esri tool. This tool generates vector service areas from a point and not a polygon; therefore, points were generated every 100 m along each PORA boundary. For PORAs with a perimeter less than 100 m, the PORA centroid was generated and for PORAs with a perimeter greater than 100,000 m, 1,000 equally spaced points were generated. The vector service areas for each PORA boundary point were merged and dissolved into a single service area for each PORA.

PORA-WAS were also determined for each of the randomly selected PORAs following the methods in Section 2.2 with the exceptions that no impeded features were used, and all roads were considered walkable. This was done to better match the vector-based approach where no walking impediments were included, and roads were not filtered based on walkability. We tested five values for the Expand Raster Value parameter: 0, 3, 5, 8, and 10 pixels.

We compared total area and spatial congruency between each PORA’s vector service area and WAS. Congruency was measured as the ratio of the spatial intersection area and the spatial union area of the PORA-WAS and vector service area. Area within the PORA boundary was excluded from both the PORA-WAS and vector service areas. Congruency ranges from zero, with no overlap between the two features, and one, identical features. We also compared these values between PORAs that were close (<20 m) to our walkable road network and those that were not. Wang et al., (2021) used 20 m as a reasonable distance to assume that a road that ended at or was adjacent to a park provided access.

## 3. Results

### 3.1. PORA assessment for CONUS

We found that, not surprisingly, access and availability of PORA space ranged across geographies and population density. Examples ranged from areas with no accessibility, accessible areas with limited availability, and areas well over any recommended threshold of availability for every community setting in CONUS.

#### 3.1.1. Summarized results

Of the resolution 9 H3 hexagons, approximately 64 % have a population greater than zero. For populated hexagons, the average population size is 6.9 people with a standard deviation of 45.8. We estimate 157,046,153 people in the CONUS (47.7 % of the CONUS population) do not live within an 800 m walk of a PORA (Table 3). However, this population is distributed across areas with wide ranging population densities. Although access to PORAs is important for all people and having PORAs within walking distance is desirable, it is less practical in sparsely populated rural areas where people are more dispersed. People living in these areas may also have incidental access to non-publicly accessible forms of outdoor recreation space. When considering only those who reside in more developed environments, the percentage of people beyond walkable access drops considerably; 13.8 % of people in areas *with* ≥ 70 % imperviousness and 5.4 % of people in areas with ≥ 85 % imperviousness (Table 3). Only 34.8 % of the CONUS population meet the LEED suggestion of at least 11.25 square meters per person, and 32.2 % and 25.0 % of the population meet the LEED minimum in areas with ≥ 70 % imperviousness and with ≥ 85 % imperviousness, respectively (Table 3).

#### 3.1.2. Example case studies

**Fig. 5.**
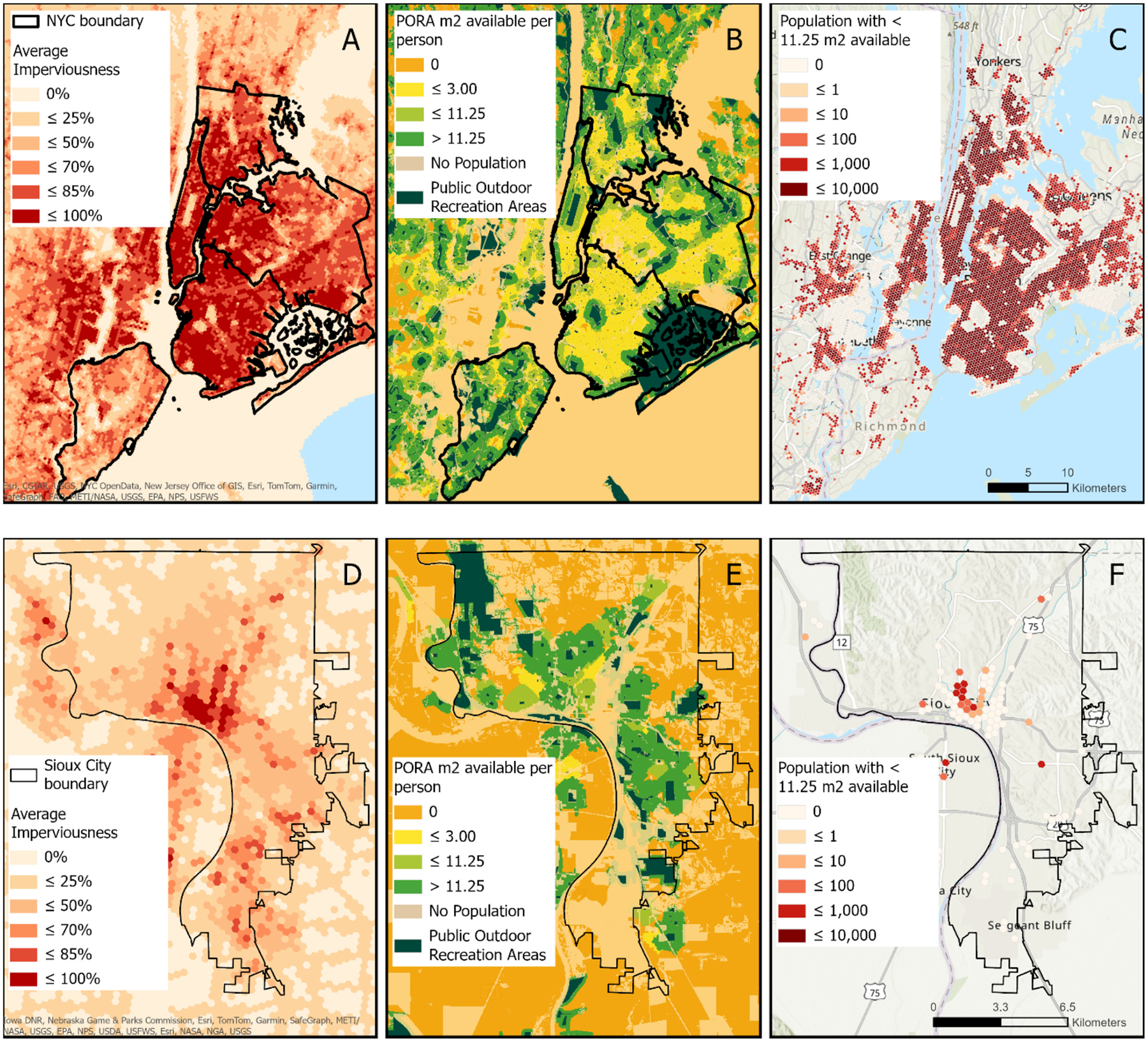
Results for New York, New York (A – C) and Sioux City, Iowa (D – F). Maps A and D show the average imperviousness summarized by H3 level-9 hexagon. Maps B and E show the square meters of PORA available per person. Maps C and F show the population with less than 11.25 square meters available per person summarized for hexagons with greater than 70% average imperviousness.

Fig. 5 displays illustrative results for New York, NY and Sioux City, IA. These maps illustrate how this method can be applied and be useful for two very different cities.

### 3.2. Metric validation

#### 3.2.1. Comparison to other measures

For two counties in California, our PORA-WAS estimates were 23 % (Orange County) and 1 % (San Francisco County) smaller than California Parks for All’s 0.5 mile buffer area (Table 4). The lower numbers were expected because of the smaller area derived by using walkable routes from a PORA versus using a radius (Fig. 3). Alternatively, our PORA-WAS estimates were between 1–17 % larger than ParkServe areas within a 10-minute walk (Table 4) likely because of the additional area of PORAs identified in PAD-US-AR. PORA area was higher than California Parks for All park area in both counties and higher for three of the four ParkServe park areas. New York City’s ParkServe’s park area was slightly more than the PORA area from PAD-US-AR (Table 4).

#### 3.2.2. Comparison of PORA-WAS and vector service areas

**Fig. 6.**
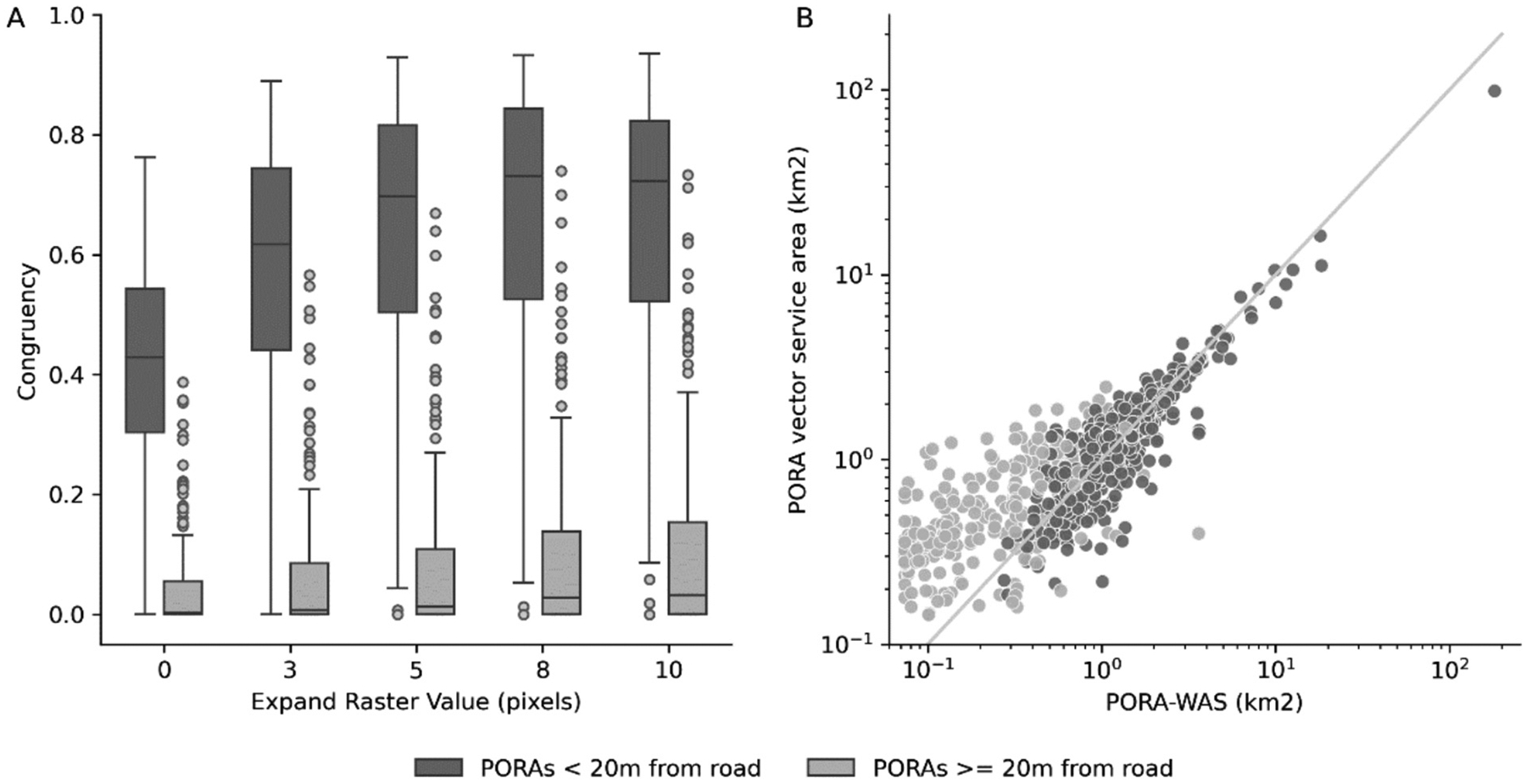
Comparisons of vector-based service areas and cost raster-based PORA-WAS for 972 random PORAs. (A): Distribution of congruency between vector-based service areas and PORA-WAS grouped by different values for the Expand Raster Value parameter. (B): Comparison of total area of vector-based service areas and PORA-WAS produced with an Expand Raster Value of 8 pixels. Note: PORA area was excluded from all measures of congruency and total area. Diagonal line represents 1:1 relationship.

**Fig. 7.**
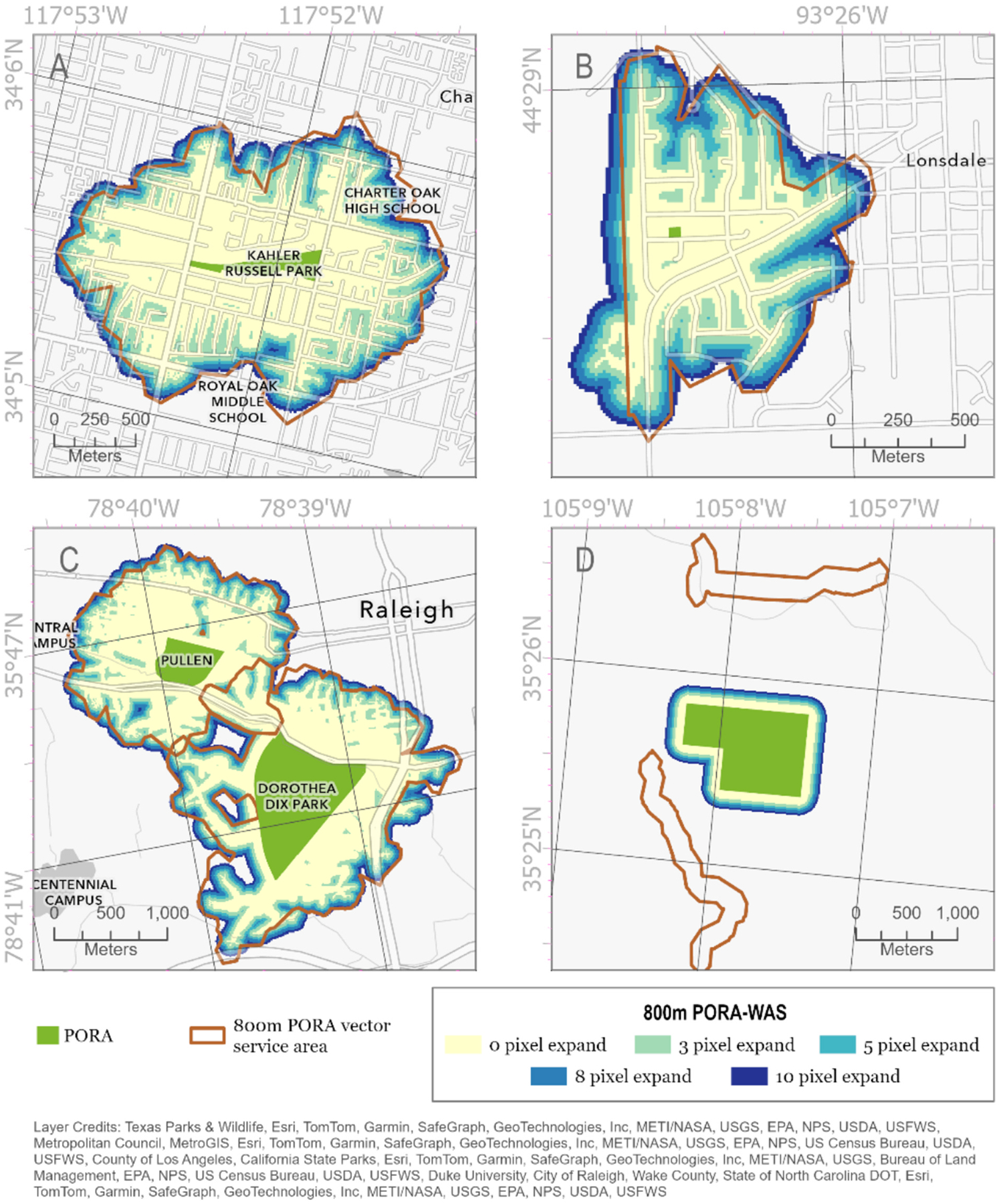
Four illustrative examples of vector-based service area and cost raster-based PORA-WAS. The figures above represent comparisons for: (A) a dense urban area outside Los Angeles, CA, (B) a smaller exurban town in Minnesota, (C) areas with overlapping PORA service areas in Raleigh, NC, and (D) a remote PORA with no digitized road access in New Mexico.

Of 1,000 randomly selected PORA boundaries, 28 were too small or irregularly shaped to be rasterized using our cost-raster method. These were almost exclusively tiny slivers or small non-adjacent pieces of larger PORAs. Median congruency for the five Expand Raster Values we tested (0, 3, 5, 8, and 10 pixels) ranged from 33.7 % to 59.7 % (Fig. 6A). Using an 8-pixel Expand Raster Value produced the highest median congruency at 59.7 % (n = 972) when compared to the vector-based service area (Fig. 6A). This number was markedly higher when only considering PORAs within 20 m of a digitized road (73.1 %, n = 714). Fig. 6B shows the relationship between PORA-WAS and the vector-based service area with those PORAs < 20 m from a digitized road clearly demonstrating closer agreement than those >= 20 m. Fig. 7 shows four examples of the vector-based service areas and our PORA-WAS. The congruency between the vector service areas and PORA-WAS developed with an 8-pixel Expand Raster Value in these examples are (A) 90.0 % for Kahler Russell Park, outside Los Angeles, CA, (B) 81.8 % for an unnamed park in Lonsdale, MN (a small exurban town), (C) 90.3 % for Pullen and 83.9 % for Dorthea Dix Parks in Raleigh, NC, and (D) 0 % for a remote PORA with no known road access in New Mexico.

## 4. Discussion

Our method was developed to measure access and availability to areas where people can engage in outdoor recreation activities. We applied our method to create a metric assessing PORA access and availability within an 800-meter walking distance of where people live.

### 4.1. PORA accessibility and availability metric

Our method offers improvements when compared to other available methods quantifying access to outdoor recreational spaces. Our method captures PORA accessibility and availability rather than measuring one or the other. Accessibility is measured using the distance along routes rather than by using Euclidean distance. Additionally, our method accounts for population density allowing it to account for PORAs that are undersized for the populations they serve and for the combined availability of multiple PORAs serving the same people.

Other methods tend to focus on determining the areas of a city within walking distance of PORA which subsequently classifies people living in those areas as either having access or not (Trust for Public Land, 2023; U.S. Environmental Protection Agency, 2015). While this strategy does identify areas in need of PORA development, it ignores the availability of PORAs relative to the population density within each PORA’s area of service. Our metric identified 57.7 million people who are within walking distance of a PORA but do not meet 11.25 m2/person LEED guidelines (Table 3). Other existing methods do provide estimates of availability, but they do not consider overlapping service areas (California Department of Parks and Recreation, 2022). Overlapping service areas are particularly relevant in dense urban areas that are highly walkable and boast numerous small PORAs. Our metric accounts for those living in areas with multiple PORAs within walking distance where people can naturally disperse among their options depending on fluctuating demand.

We were specifically interested in examining PORA access and availability within walking distance. Typically, park access can be estimated by either Euclidean or network distances. Zhang et al., (2011), and Liu et al., (2022) also assessed availability and accessibility but used Euclidean distance rather than network distance. We used an efficient raster network distance approach and assigned walkability using an innovative combination of road/pathway routes and water bodies. We intentionally identified barriers to walking (e.g., water bodies, highways, exit/entrance ramps) and incorporated these into our walkability cost raster.

While our method may readily be scaled up to a driving distance instead of walking distance, we believe it is crucial to understand how accessibility and availability of PORAs function at the scale of a person walking from their home to a PORA. In that same context, we chose the dasymetric population raster to model population density at a pedestrian scale rather than considering population summarized by a US Census reporting unit.

Our methodology and resulting metric is consistent and continuous across a study area and can highlight PORA needs in any sized community. The limited number of inputs (i.e., roads, PORAs, population) allow for use in data-sparse communities while also allowing data-rich communities to use more refined inputs (e.g., sidewalks, handicap accessibility, obstructions). Our method can easily be recalculated based on contractions and expansions of population, changes in pedestrian networks, or development of additional PORAs over time.

### 4.2. Example case studies

New York City (NYC), New York and Sioux City, Iowa illustrate many of the advantages of our metric. NYC is the most densely populated city in America (U.S. Census Bureau, 2021) and sees heavy pedestrian activity. Much of the city is characterized by impervious landscapes. Without formal, planned areas like parks, city residents are unlikely to have access to any PORA. As such, it is critical that city planning be informed by appropriate data. Fig. 5B shows that NYC has a good distribution of PORAs; very few areas in the city are beyond walking distance of a PORA. This is due not only to the walkability of the city but also the number and spatial distribution of the city’s PORAs. However, as Fig. 5C illustrates, these PORAs are often not large or numerous enough to ensure that people have ample availability. Millions of people living in NYC do not meet LEED standards of 11.25 square meters per person. While NYC is known for having one of the largest urban park systems in the United States (Jung, 2023) and the NYC Department of Parks & Recreation has impressively estimated that 84 % of residents live within walking distance of a park as of December 2024 (NYC Parks, 2024), very little can be found addressing availability. Our metric not only highlights the lack of PORAs, but can guide city planners to both the yellow areas in Fig. 5B with the least availability and the areas with the most people in need (Fig. 5C).

In Sioux City, Iowa, there is a different pattern. Unsurprisingly there is much less impervious surface in Sioux City. In Fig. 5E, we see that a greater proportion of Sioux City is beyond walking distance from a PORA than in NYC. There are both relatively fewer PORAs and a less dense road network. Where PORAs are available, they generally provide ample availability given the lower population density relative to the size of the PORA. However, Fig. 5F illustrates an important benefit of our metric to less densely populated communities. There is a small area in downtown Sioux City that appears to be both highly impervious and lacks sufficient PORAs for the immediate population in those neighborhoods. We estimate the population of this area to be 2,066, about 2.4 % of the population of Sioux City.

### 4.3. Comparison of PORA-WAS and vector service areas

We found that expanding our initial walkable area by a fixed number of pixels was a necessary step in estimating PORA-WAS. We selected eight pixels for our Expand Raster Value because this value was the best fit for our maximum distance (Fig. 6A), but this value may need to be reevaluated for distances other than 800 m. Our PORA-WAS results aligned well with the vector-based service areas approach, particularly for PORAs that were close to walkable paths (i.e., within 20 m of a digitized road). Generally, the PORA-WAS followed the same shape and area of the vector-based alternative (Fig. 6, Fig. 7) without the burden of using a commercial routing service or developing a complex network dataset of roadways. Mulrooney et al., (2017) performed a comparison at the same resolution we used (10 m) for travel times to a grocery store and found no significant difference between raster and vector based measurements.

Fig. 7D shows a common discrepancy between the PORA-WAS and vector service areas for remote PORAs (i.e., > 20 m from a digitized road). Because there is no walkable route, as we have defined them, to these remote PORAs, the PORA-WAS is effectively a small buffer around the PORA. On the other hand, the vector service areas for these areas are often no more correct. We found the vector service area begins at the nearest point along the road network regardless of the distance from the PORA to that road network. This can lead to vector service areas that are completely detached and further than our maximum walking distance from the PORA itself. More investigation is required to assess these PORAs that are not close to a road. Because our effort was focused on PORA’s within walking distance, these remote PORAs are irrelevant to our results.

## 5. Limitations

While we believe our method to be a major step in determining both PORA accessibility and availability in a single, cohesive metric, there are limitations to our application of it, especially when applying it to the CONUS scale. This analysis tends toward overestimating walkability to prioritize confidence in our identification of those who live outside a reasonable walking distance to a PORA. We used 800 m as a maximum walking distance, one of the longer distances found in the current literature. We assumed if a road is not inherently dangerous to pedestrians it should be considered a walkable route even though American roadways are frequently designed without pedestrian usage in mind. In fact, one option for communities with insufficient service to PORAs may be to increase pedestrian infrastructure rather than build new PORAs themselves. It may be that a sidewalk, or crosswalk connects an already existing PORA to a new population. If routes identified are locally known to be unfriendly to pedestrian traffic, they should be assessed for potential improvement.

This analysis was limited by the availability of CONUS wide data. There are no consistent or complete datasets for sidewalks, pedestrian paths, walkways, or trails across the United States. OpenStreet Map does include much of this information but is not nationally reliable (OpenStreetMap, 2022). Such data would greatly improve the estimate of walkable routes in these methods, and we encourage its use at a local level analysis or future country wide efforts when those data become available. While PAD-US is a valuable and unique data set, it is far from complete, and its consistency may vary across states. The PAD-US-AR dataset is already dated with new versions of PAD-US having been released. Similarly, the PAD-US-AR data rely on the accuracy of the ‘Public Access’ variable in the PAD-US which may be incorrect or missing from some protected areas. The PAD-US-AR data likely under-represents recreational areas that are not “green” like playgrounds and should not be considered a complete catalog of every PORA in the country. Fry et al. (2024) present the results of a comparison of multiple sources of park boundary definitions for Philadelphia, PA including PAD-US-AR. In Sioux City, Iowa, we are aware of at least one PORA, Pearl Street Park, that was not included in our analysis. While its inclusion would not have drastically altered the accessibility or availability estimates in our case study, we are aware that this may not be the case for every missing PORA. We are also aware that PAD-US-AR misses PORA’s on some Native American lands.

PAD-US does not report the quality, amenities offered, or usage patterns of recreational areas. It is possible that we overestimated PORA walkability in this way by including areas that exist but are under-utilized by the surrounding population because they do not provide the opportunities they need. Furthermore, this method does not consider temporal usage patterns, such as peak hours or weekends. Data derived using our method could be used in conjunction with visitation data collected via traditional means or human mobility data derived from mobile phone applications. This nascent technology is proving to be a powerful tool for assessing visitation to parks (Song et al., 2022; Tsai et al., 2023). The addition of usage data would give planners a complete picture of park access, availability, and usage.

Finally, we did not incorporate barriers along PORA edges such as fencing, because such data are impractical to obtain at a national level. While we do not identify or model individual PORA entrances, our method prioritizes routes along and across walkable roads which mimics other methods that use road/park intersections as entrances (Trust for Public Land, 2023; Wang et al., 2021). These are areas we believe are more likely to provide entry to PORAs, but these intersections are not guaranteed to provide entry. This is a significant concern, and we strongly encourage incorporating such obstacles as ‘impeded’ when applying these methods for study areas where these barriers are known. Ultimately this metric serves as a baseline measurement that can be compared in and across communities of all sizes. As with most metrics, refined data and local knowledge can inform and provide better estimates of these measurements.

### 5.1. GIS toolbox and future direction

The methods described here are available in the ATtILA toolbox, version 3.0 (U.S. Environmental Protection Agency, 2023). This toolbox is developed and maintained by EPA’s EnviroAtlas project (Pickard et al., 2015) to assist users in independent analyses using their own input data and parameters. It is our hope that local communities use these data or our toolbox along with their own PORA and walkable routes data to assess their communities’ needs. Users could also apply these methods to PORA’s with specific known qualities (e.g., water features, playgrounds) or usage patterns. Planners could use our methods to conduct area-wide parks and recreation assessments along the lines of a robust assessment conducted by the Los Angeles County Department of Parks and Recreation (2022). This assessment considered the needs of different sectors of the population (e.g., rural versus urban) as well as park amenities (e.g., trails, water amenities).

With our toolbox, users can specify how far pedestrians are willing to walk, how difficult it is for pedestrians to cross different landscape types and set their own thresholds for how much space per person is adequate. Increasing PORA access may include adding new recreational areas or improving walkability to existing infrastructure. The raster layer produced by our methods can help identify PORAs that have limited pedestrian access versus areas with excellent walkability but proportionally little PORA availability.

The cost raster development and weighting scheme offers numerous opportunities for refinement. For example, D’Orso and Migliore (2020) proposed a suite of walkability indicators (e.g., including slope, surface condition, streetlights, retail shops) that could be incorporated and Hollenstein and Bleisch (2016) applied different weights to streets based on speed limit, number of crossings, and number of sidewalks. While this level of detail was not practical for a CONUS effort, local efforts could take advantage of these schemes.

We intend to test and adapt our cost raster methods, if necessary, to measure access to PORAs by personal vehicle or public transportation for multiple travel distances. PORAs within different driving distances provide their own suite of benefits that may be different to a park within walking distance (e.g., scenery, wildlife viewing, adventure, and opportunities for a particular type of activity like kayaking or skiing). It would be worthwhile to estimate how many people live within different driving distances of PORAs across the country and how well such PORAs are equipped to meet the demand of visitors.

## 6. Conclusion

We developed an innovative raster-based method to measure PORA access and availability and coded this method into ATtILA, an easy-to-use ArcGIS Pro Toolbox. The tool requires few inputs and is flexible to user needs. We believe this toolbox will be useful to local, county, and state park planners and managers, advocacy groups, and researchers interested in understanding patterns of access and availability. We applied our method to assess PORA access and availability within walkable distance which we defined as 800 m. Our results compared favorably against other efforts that assess PORA access or availability. Benefits of our method over others is that it measures both access and availability, distances are based on travel along a transportation network rather than Euclidean distance, and it accounts for overlapping PORA service areas.

We also conclude that we need better data defining and describing outdoor recreation areas. While PAD-US-AR is the best source we know of to identify PORAs in the U.S., we know that many PORAs are missing and that it contains inconsistencies.

## Figures and Tables

**Table 1:** Datasets used to measure PORA accessibility and availability across the conterminous United States.

Dataset/version/data type	Source	Method Use
PAD-US-AR Version 1; 10-31-2022, Vector — polygon–	Protected Area Database US — Accessible Recreation	PORA boundaries
National Hydrography Dataset Plus High Resolution (NHD Plus HR) Release 1; 2021, Vector - polygon and polyline	USGS	Impeded routes
ArcGIS StreetMap Premium North America 2021 Release 1, Vector — polyline	ESRI	Walkable routes; Impeded routes
2020 Dasymetric Population for the Conterminous United States v1.1 – Raster 30 *m*	Environmental Protection Agency: EnviroAtlas	Population

**Table 2:** Parameters used with the ATtILA Create Walkability Cost Raster utility and Pedestrian Access and Availability Tool to measure PORA accessibility and availability for CONUS.

ATtILA Parameter	Value
Maximum Travel Distance	800 *m*
Walkability Cost Raster Cell Size	10 *m*
Walkable Cost Raster Value	1
Background Value	10
Expand Raster Value	8 pixels

**Table 3:** Number of people in CONUS with access and availability of PORAs within an 800- meter walk from their homes summarized for H3 level-9 hexagons. All counts are rounded to the nearest 100,000 and reported in millions. Population counts determined from 2020 EPA dasymetric population. Percentages are of the total population listed in the first row of each column.

	All Hexagons within CONUS		Hexagons ≥ 70 % Imperviousness		Hexagons ≥ 85 % Imperviousness	
	Count	Percent of Total	Count	Percent of Total	Count	Percent of Total
Total Population	329.3		35.5		9.5	
Population with Access and ≥ 11.25 m2 Available	114.5	34.8 %	11.4	32.2 %	2.4	25.0 %
Population with Access and (≥ 3 m2 and < 11.25 m*2* Available)	36.5	11.1 %	8.9	25.2 %	2.3	23.9 %
Population with Access and < 3 m*2* Available	21.2	6.4 %	10.2	28.8 %	4.3	45.7 %
Population with No Access	157.0	47.7 %	4.9	13.8 %	0.5	5.4 %

**Table 4:** Comparison between California Parks for All 0.5-mile buffer areas, ParkServe’s area within 10-minute walk, and our PORA-WAS estimates of 800 m walk from PAD-US-AR. For this comparison, Park/PORA areas were excluded from all service area estimates.

		California Parks for All		ParkServe		PORA-WAS	
County	Total Area (km^2^)	Park Area (km^2^)	Area within 0.5 miles (~804 m) of a park (km^2^)	Park Area (km^2^)	Area within 10-minute walk of a park (km^2^)	PORA Area (km^2^)	Area within 800 m walk of a PORA (km^2^)
New York City, NY	783	na	na	135	585	130	606
Sioux City, IA	155	na	na	5.8	49	15	55
Orange County, CA	2062	452	1158	477	828	580	919
San Francisco County, CA	123	23	96	25	94	25	95

## Data Availability

The ATtILA for ArcGIS Pro toolbox is available from EnviroAtlas (https://www.epa.gov/enviroatlas/attila-toolbox) or EPA’s GitHub repository (https://github.com/USEPA/ATtILA2; doi: 10.5281/zenodo.8048193). Detailed usage instructions are available at the GitHub Wiki (https://github.com/USEPA/ATtILA2/wiki). PORA Access and Availability raster at 10 m resolution for CONUS can be accessed and viewed on EPA’s EnviroAtlas (https://www.epa.gov/enviroatlas).

## HIGHLIGHTS

- New GIS toolbox to assess availability and accessibility of outdoor recreation.
- Raster method to identify service areas was comparable to vector alternative.
- Assessed walkable outdoor recreation opportunities for conterminous U.S.
- 21.2 million people living near a park have limited public space available.
